# Cardiac Exosomes in Ischemic Heart Disease—A Narrative Review

**DOI:** 10.3390/diagnostics11020269

**Published:** 2021-02-09

**Authors:** Øystein Røsand, Morten Andre Høydal

**Affiliations:** Group of Molecular and Cellular Cardiology, Department of Circulation and Medical Imaging, Faculty of Medicine and Health, Norwegian University of Technology and Science (NTNU), 7030 Trondheim, Norway; oystein.rosand@ntnu.no

**Keywords:** cardiac exosomes, coding and non-coding RNA species, cardioprotection

## Abstract

Ischemic heart disease (IHD) is the primary cause of death globally. IHD is associated with the disruption of blood supply to the heart muscles, which often results in myocardial infarction (MI) that further may progress to heart failure (HF). Exosomes are a subgroup of extracellular vesicles that can be secreted by virtually all types of cells, including cardiomyocytes, cardiac fibroblasts, endothelial cells, and stem and progenitor cells. Exosomes represent an important means of cell–cell communication through the transport of proteins, coding and non-coding RNA, and other bioactive molecules. Several studies show that exosomes play an important role in the progression of IHD, including endothelial dysfunction, the development of arterial atherosclerosis, ischemic reperfusion injury, and HF development. Recently, promising data have been shown that designates exosomes as carriers of cardioprotective molecules that enhance the survival of recipient cells undergoing ischemia. In this review, we summarize the functional involvement of exosomes regarding IHD. We also highlight the cardioprotective effects of native and bioengineered exosomes to IHD, as well as the possibility of using exosomes as natural biomarkers of cardiovascular diseases. Lastly, we discuss the opportunities and challenges that need to be addressed before exosomes can be used in clinical applications.

## 1. Ischemic Heart Disease

Ischemic heart disease (IHD) is the main global cause of death, accounting for >9 million deaths in 2016 according to the World Health Organization (WHO) estimates [1]. IHD is most often associated with total or partial disruption of blood supply to the heart muscles, which results in the consequent shortage of oxygen and nutrients to the cardiac cells, which commonly causes myocardial infarction that eventually may progress to the development of HF. As a global health problem, heart failure (HF) affects approximately 26 million people annually, resulting in more than 1 million hospitalizations in Europe and the US [2,3,4]. Although HF can be caused by many cardiovascular conditions, the predominant cause of HF in the western world is IHD [5]. The extent of cell dysfunction, injury, and/or death of an ischemic incident is influenced by its magnitude and duration. Both in animal and human models, irreversible cardiomyocyte damage occurs after 20 min of ischemia [6]. The mechanisms contributing to ischemic injury are multifactorial, with lack of oxygen and essential nutrients causing metabolic acidosis, Ca^2+^ overload, and accumulation of reactive oxygen species (ROS) [7,8,9] as central components. Mitochondrial Ca^2+^ overload triggers mitochondrial depolarization, swelling, and opening of the mitochondrial permeability transition pore (mPTP) [10,11,12], thus leading to cell death [13,14], and consequently, a buildup of dead cardiomyocytes in the ischemic regions. After an ischemic incident, the reestablishment of blood flow is indispensable to salvage ischemic tissue. The surgical procedure commonly used to restore proper blood flow to the heart, primary percutaneous coronary intervention (PCI), or thrombolytic therapy, triggers a phenomenon called ischemia reperfusion (IR) [15]. The process of restoring blood to the ischemic myocardium may, however, paradoxically further induce tissue injury. This phenomenon is aptly named ischemia reperfusion injury (IRI), and is associated with mitochondrial dysfunction, increases in oxygen free radical production, hypercontracture, and myocardial inability to readjust to aerobic metabolism, culminating in the loss of viable myocardium [14,15]. While reperfusion therapies are some of the most successful discoveries in medicine, cardioprotective therapies targeting reperfusion-related injury have not yet been successful and remain a major unmet clinical challenge in cardiology [16]. Attention has therefore turned to adjunctive pharmacologic treatments to enhance myocardial tolerance during and after myocardial infarction.

## 2. Remote Ischemic Preconditioning

**Figure 1:**
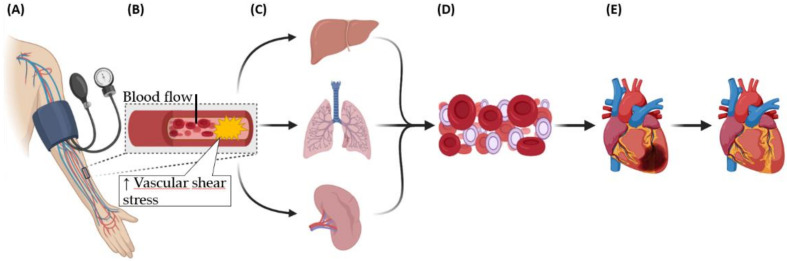
Schematic overview of remote ischemic preconditioning (RIPC) stimulating exosome production resulting in myocardial protection. (**A**) Repeated cycles of ischemia reperfusion induced by blood pressure cuff inflation at the arm. (**B**) RIPC induces shear stress in the vasculature. (**C**) The shear stress stimulates the humoral system to release cardioprotective factors into the blood. (**D**) Cardioprotective factors are carried by the blood. (**E**) Cardioprotective factors reach the myocardium and activate cardioprotective signaling pathways that lead to reduced tissue damage and improved function.

Because of the impact of ischemia and IRI on human health, novel therapeutic strategies are required to preserve myocardial function and prevent the transition to HF. One strategy that has received significant attention during the last decades is remote ischemic preconditioning (RIPC) (Figure 1) [17,18].

RIPC is a phenomenon whereby cardioprotection is obtained from the application of shorter periods of ischemia reperfusion in distant organs, e.g., the upper limb, before the onset of a myocardial ischemic event [19]. In earlier studies, RIPC has been demonstrated to reduce ischemic myocardial damage in patients undergoing cardiopulmonary bypass (CPB) surgery [20,21] and during coronary artery bypass grafting (CABG) [19]. Furthermore, RIPC has been demonstrated to better preserve myocardial adenosine triphosphate (ATP) levels and reduce production of ROS during IRI [22,23]. Moreover, in 2014, our group published a paper indicating that RIPC protects the mitochondrial function from IRI during CABG surgery [24], which supports earlier data that designates the mitochondria as key players mediating the cardioprotective effects of RIPC [25,26]. However, the cardioprotective effects are lost if RIPC is not followed by reperfusion within certain time intervals. Furthermore, the use of RIPC is not possible when the ischemic event is unpredictable, e.g., acute myocardial infarction (AMI), and can thus only be used in patients undergoing elective procedures as earlier mentioned, i.e., CPB and CABG [27].

**Figure 2:**
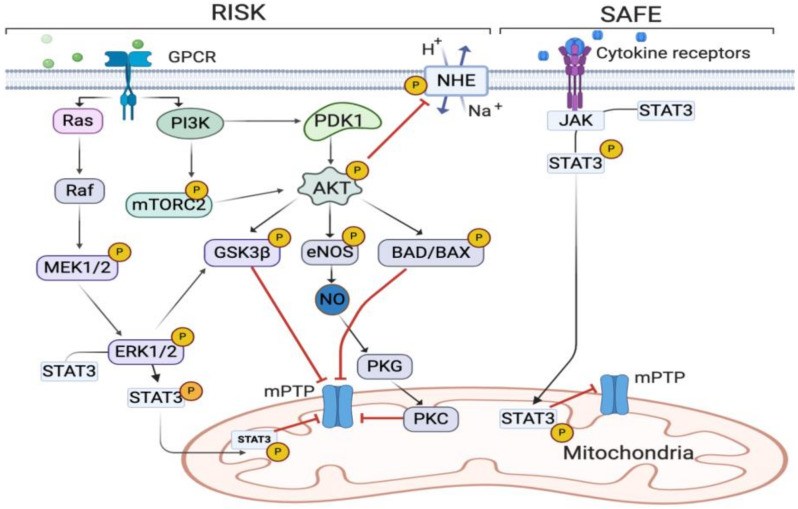
Simplified overview of major protective signaling pathways involved in RIPC, the reperfusion injury salvage (RISK) pathway, and the survivor activating factor enhancement (SAFE) pathway. Abbreviations: G protein-coupled receptors (GPCRs); MEK1/2, also known as mitogen-activated protein kinase 1/2; Erk1/2, extracellular-regulated kinases 1/2; PI3K, phosphatidylinositol-4, 5-bisphosphate3-kinase; Bcl-2-associated death promoter (BAD); Bcl-2-associated X protein (BAX); glycogen synthase kinase-3ß (GSK3ß); mitochondrial permeability transition pore (mPTP). Akt, also known as protein kinase B; nitric oxide synthase 3/endothelial nitric oxide synthase (NOS3/eNOS) and nitric oxide (NO); Janus kinase (JAK), protein kinase G (PKG); protein kinase C (PKC), mechanistic target of rapamycin (mTOR); sodium hydrogen exchanger (NHE); signal transducer and activator of transcription 3, STAT-3. P indicates phosphorylation. Red lines indicate inhibition.

Recent studies have shown that the intermediate hypoxia in remote organs, caused by RIPC, activate hypoxia-inducible transcription factor 1α (HIF-1α), which activates the adaptive response to hypoxia [28,29]. HIF-1α activation caused by RIPC has been shown to accelerate cognitive functional recovery after brain ischemia in mice [29]. Moreover, a study published by Pan T et al. in 2019 proved that RIPC is able to block the elevation of tumor necrosis factor α (TNF-α) and interleukin 6 (IL-6) levels in serum. The same study showed that RIPC could reduce cell apoptosis by suppressing levels of B-cell lymphoma 2 (BCL-2) and cleaved caspase-3 [30]. Furthermore, RIPC can activate the reperfusion injury salvage (RISK) pathway. RISK refers to the pro-survival kinase Akt, a downstream target of phosphoinositide 3-kinase (PI3K) signaling [31,32]. Several studies show potential for increased phosphorylation of Akt in mediating cardioprotection after ischemia [33,34,35,36], via the inhibition of the Bcl-2-associated death promoter (BAD), Bcl-2-associated X protein (BAX) [37], and glycogen synthase kinase-3β (GSK3β) [38] that causes cell-death via the opening of the mitochondrial permeability transition pore (mPTP). Akt also activates the nitric oxide synthase 3/endothelial nitric oxide synthase (NOS3/eNOS) pathway to synthesize nitric oxide (NO) that also hinders the opening of the mPTP [39]. Furthermore, Akt phosphorylates and inactivates the sodium/hydrogen exchanger (NHE) and thereby prolongs the acidotic period after ischemia [40]. Likewise, RIPC can activate the survivor activating factor enhancement (SAFE) pathways. The SAFE pathways refer to the activation of Janus kinase (JAK) and upstream activation of STAT3 (signal transducer and activator of transcription 3). STAT-3 is present in mitochondria, where it modulates respiration and inhibits the opening of the mPTP. Besides being activated by TNF-α, the SAFE pathway is activated by neuregulin-1 (NRG-1). This upstream signaling complex consists of the growth factor NRG-1 and its tyrosine kinase receptor, ErbB. Activating NRG-1-ErbB was recently shown to provide a viable strategy for treating HF by stimulating the proliferation of cardiomyocytes (CMs) [41]. In CMs, NRG-1 activation reduced apoptosis after hypoxia-reoxygenation and intravenous NRG-1 administration reduced myocardial infarct size following IR [42]. Additionally, RIPC can help increase the concentrations of vascular endothelial growth factor in plasma and therefore reduce the severity of paraplegia following spinal cord injury (SCI) [43]. Interestingly, in vivo experiments in the Zucker fatty rat model have shown that RIPC does not seem to carry the same protective effects of RIPC to the myocardium in type 2 diabetes [44]. One of the major findings in this study was that diabetes was associated with a defective humoral communication linked to the composition of exosomes released from distant organs [44]. The blunted effect of RIPC has also been observed in patient studies of diabetes [45]. The complete underlying mechanism responsible for the loss of cardioprotection in diabetics remains unresolved. However, there are several lines of evidence indicating that diabetes also interrupts vital intracellular signaling processes that mediate the beneficial effect of ischemic conditioning that causes cardioprotection (reviewed in [46]). In order to ensure proper supply of oxygen and nutrients to the blood deprived areas, cells and organs must communicate. The mechanism that mediates and transfers inter-organ cardioprotection remains undefined [47]. Typically, intercellular communication can occur directly between adjacent cells via gap junctions [48], or indirectly at longer distances through soluble factors and extracellular vesicles (EVs) [49]. There are several lines of evidence showing that the cardioprotection by RIPC is carried out by a sub-group of EVs named exosomes. In the following sections, we further discuss the benefits of exosomes and their cardioprotective effects connected to IHD and RIPC. An overview of the common signaling pathways involved in RIPC is displayed in Figure 2.

## 3. Exosomes

The International Society for Extracellular Vesicles (ISEV) classifies EVs as “the generic term for particles naturally released from the cell that are delimited by a lipid bilayer and cannot replicate”. Unless authors can establish specific markers of subcellular origin that are reliable within their experimental system(s), ISEV urges to consider the use of operational terms for EVs [1]. In this review, when referring to “exosomes”, we refer to a subgroup of EVs ranging in size from 30–150 nm [50].

Exosomes were first described in 1977 under the name prostasomes [51], and were given the name exosome in 1987 [52]. Exosomes have been identified in all body fluids, including serum, plasma, amniotic fluid, saliva, breast milk, urine, and in cell culture media, and are secreted by all cells [53,54]. Initially, exosomes were believed to be a way for the cells to discard waste [52], but are now regarded as a well-regulated form of intercellular communication. Exosomes are generated from the invagination of endosomes resulting in the formation of multivesicular bodies (MVBs), which then are secreted through fusion with the cell membrane into the extracellular space. They function as biological vehicles, transferring information between donor and target cells [55,56,57]. The composition and cargo of the exosomes change depending on the cell type of origin and different cellular conditions or treatments. Furthermore, exosomes have been shown to carry proteins, lipids, coding and non-coding RNA, and DNA [58,59,60]. In addition, exosomes are enriched in lipids, such as cholesterol, phosphatidylserine, sphingomyelin, glycosphingolipids, and ceramide. These are conserved and essential for maintenance of exosome morphology, exosome biogenesis, and regulation of homeostasis in target cells [61]. The precise selection of exosomal cargo is not yet completely understood, although studies indicate that exosome formation and protein sorting can be managed by the endosomal sorting complexes required for transport pathway (ESCRT) [62,63] or lipid raft-mediated pathway [64]. ESCRT is comprised of four complexes; ESCRT-0, ESCRT-I, ESCRT-II, and ESCRT-III, with multiple associated proteins, e.g., Vacuolar protein sorting-associated protein 4, Tumor susceptibility gene 101 protein, and programmed cell death 6-interacting protein [65]. The lipid bilayer of the exosomes protects their cargo, allowing them to persevere in the extracellular environment. Once secreted, exosomes travel into the bodily fluids to enter neighboring or distant target cells. It has been shown that target cells internalize exosomes through a variety of methods, e.g., ligand receptor binding, endocytosis, or membrane fusion [66].

## 4. Exosomes Derived from Cardiac Cells

**Figure 3:**
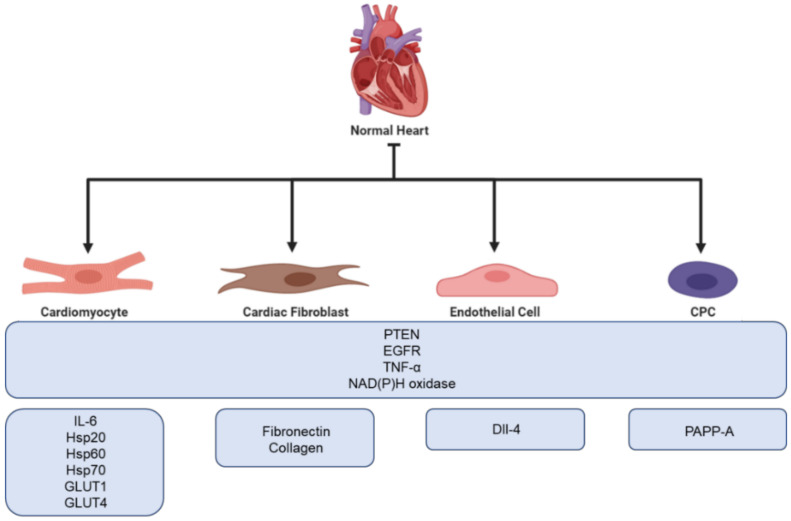
Overview of the cardioprotective proteins found in exosomes secreted from different cells of the myocardium.

Recently, the cardiovascular field has had an increasing interest in exosomes due to their aforementioned cardioprotective possibilities. Studies have proven that exosomes can carry many proteins that are relevant for cardioprotection, e.g., phosphatase and tensin homolog (PTEN), annexins, epidermal growth factor receptor (EGFR), TNF-α, and NAD(P)H oxidase [67]. Although cardiac cells are not considered typical secretory cells, a number of in vitro experiments using rodents have indicated that cardiomyocytes secrete exosomes both in healthy and ischemic conditions, which mediate communication between healthy and damaged cells [68]. This was first proven in 2007, where exosomes were showed to be released both in physiological and hypoxic conditions [69]. Exosomes derived from cardiomyocytes have been shown to modulate cell proliferation, migration, differentiation, survival, and angiogenesis in response to ischemic incidences [70,71]. There is an increasing number of observations showing that cardiomyocyte-derived exosomes are enriched with inflammatory factors such as TNF-α and IL-6 [72,73]. Additionally, studies have demonstrated that cardiomyocyte-derived exosomes contain multiple heat shock proteins (Hsp20, Hsp60 and Hsp70) [69,74,75]. Furthermore, exosomes derived from cardiomyocytes have been found to carry functional glucose transporter proteins (GLUT1, GLUT4) and glycolytic enzymes (lactate dehydrogenase) [74]. An overview of proteins found in exosomes is presented in Figure 3. In addition to cardiomyocytes, both cardiac fibroblasts, endothelial cells, and Cardiosphere-Derived Cells (CDC) has been shown to secrete exosomes.

A total of 60%–70% of all normal cardiac cells are made up of cardiac fibroblasts, which is one of the main building blocks of the extracellular matrix (ECM) [75]. During ischemia, cardiac fibroblasts become activated and involved in cardiac fibrosis and remodeling. Furthermore, with their secretory activity, they can influence the physiology of other cardiac cells [76]. Thus, fibroblasts play a crucial role in cardiac repair. However, excessive cardiac fibrosis is a major problem in most types of heart disease as it can interfere with normal heart function [77]. Proteomic analysis of cardiac fibroblasts from neonatal rats has revealed that cardiac fibroblast secreted exosomes are upregulated under hypoxic conditions. Furthermore, hypoxia has been proven to promote cardiac fibroblast exosomal enrichment in ECM proteins, e.g., fibronectin and collagen, as well as multiple mitochondrial associated proteins. This might indicate that cells use exosomes to modulate dysfunctional mitochondria during hypoxic stress [78]. Moreover, Abrila et al. [79] reported that the administration of cardiac fibroblast derived exosomes results in a 25% reduction in infarct size in a rat model of myocardial infarction compared to controls. Additionally, the presence of cardiac fibroblast derived exosomes in co-culture increased the viability of neonatal rat cardiomyocytes following hypoxia-reoxygenation injury. Furthermore, Hui et al. [80] have shown that cardiac fibroblast derived exosomes possess protective functions in cardiomyocytes both during acute myocardial infarction and ischemic post conditioning through the microRNA (miR)-423-3p/RAP2C pathway.

Endothelial cells form the endothelial barrier between blood and surrounding tissue and play a crucial role in the maintenance of cell homeostasis. When exposed to stress or damage, endothelial cells secrete cytokines, growth factors, and exosomes [81]. The cargo of endothelial exosomes has been shown to change under different conditions, e.g., hypoxia, often resulting in higher expression of proteins involved in ECM remodeling. Endothelial cells use exosomes for communication and to facilitate angiogenesis. Delta-like 4 factor (DII-4) has been identified in endothelial cell exosomes, promoting the increase in angiogenesis by inhibiting notch signaling. A study performed by Amabile et al. showed that surface biomarkers on endothelial exosomes correlate with cardiometabolic risk factors, e.g., CD144^+^ expression is significantly upregulated in patients suffering from hypertension [82]. Additionally, endothelial exosomes have been proven to play a role in the prevention of atherosclerosis through the Krüppel-like factor 2 (KLF2)-miR-143/145 pathway [83].

Cardiac-derived progenitor cells (CPCs) are a group of heterologous cells capable of responding to injuries and differentiating into new cardiac cells. If cultured in suspension, CPCs grow as spherical aggregates aptly named cardiospheres. It has been proven that both CPCs and cardiospheres are able to release exosomes possessing cardioprotective capacities. A recent study suggests that the presence of pregnancy-associated plasma protein-A (PAPP-A) on the surface of CPC exosomes aid in their cardioprotective capacity. PAPP-A functions by cleaving insulin-like growth factor binding protein-4 (IGFBP-4), which prompts the release of Insulin-like growth factor 1 (IGF-1), a key cardioprotective factor [84,85,86]. Furthermore, to investigate if exosomes released from CPC during hypoxia were able to protect the heart during in vivo IRI conditions, Gray et al. injected rats undergoing IR performed by ligation of the coronary with hypoxic derived CPC exosomes. They found that injection by CPC exosomes improved cardiac function by enhancing the tube formation of endothelial cells and decreased profibrotic gene expression resulting in delayed fibrosis [87].

## 5. The Reported Genomic Cargo of Cardiac Cell Derived Exosomes

Exosomes are also capable of transferring genetic materials, i.e., different types of RNA molecules, including microRNA (miR), messenger RNA (mRNAs), long non-coding RNA (lncRNA), and circular RNA (circRNA). Among the genetic materials detected in exosomes, miRs have received considerable attention in the context of cardiovascular disease. miRs are endogenous, short (17–25 nucleotides), and highly conserved non-coding RNA molecules, with key functions of fine-tuning gene expression by interfering with the translation of specific mRNAs at the post-transcriptional level [88]; hence miR are important components in the pathogenesis of heart failure as well as adaptive and maladaptive cardiac remodeling.

Recently, several specific circulating miRs have been associated with the development of HF. Reduced levels of circulating miRs, e.g., miR-18a, miR-27a, miR-30e, miR-26b, miR-199a, miR-106a, miR-652, let-7i, miR-18b, miR-18a, miR-223, miR-301a, miR-652, and miR-423 have been found in patients with HF, whereas the development of HF following ischemia has been associated with increased levels of miR-1, miR-133, miR-21, miR-29b, miR-192, miR-194, miR-34a, miR-208, miR-499, miR-423, miR-126, miR-134, miR-328, and miR-486, and decreases in miR-106, miR-197, and miR-223 (reviewed in [89]). Likewise, the levels of miR-144 have been found to increase by 1.6-fold in healthy human subjects undergoing a RIPC protocol [90]. In addition, a recent study performed on a large-scale study that included two independent cohorts of 2203 HF patients found that increased levels of miR-1254 and miR-1306 were associated with increased risk of death and hospitalization [91].

Studies on exosome secretion show that exosomes released from cells exposed to hypoxia are enriched with specific miRs, more specifically miR-126 and miR-210 [92]. Furthermore, a study performed by Matsumoto et al. on post myocardial infarction patients reported that serum levels of p53-responsive miRs (including miR-192, miR-195, and miR-34a) were significantly higher in the exosome fractions from HF patients than those of the control group [93]. Many studies have identified specific cardiomyocyte related genetic material within secreted exosomes. Wang et al. demonstrated high levels of miR-320 in exosomes secreted from cardiomyocytes of diabetic patients [94]. Another study has found that exosomes from cardiomyocytes are enriched with miR-29b, miR-323-5p, miR455, and miR-466 [95]. Additionally, miR-27a, miR28-3p, miR-34a, and miR-208a have been found to be highly expressed in cardiomyocytes and preferentially incorporated into exosomes [96,97]. It is worth mentioning that miR markers have also been identified in exosomes derived from other cells of the heart, e.g., cardiac fibroblast (miR-21* [98]) and endothelial cells (miR-143 and miR-145 [83]). An overview of miR in ischemic heart disease found is presented in Table 1.

lncRNAs are >200 nucleotides long non-coding transcripts, which are essential for the regulation of tissue homeostasis. The human genome has been found to contain over 50,000 lncRNAs, located within introns or antisense transcripts of coding genes, overlapping exons of coding genes or their promoters, or between genes [100]. Some lncRNAs have been shown to regulate the expression of coding genes at both the post-transcriptional and transcriptional level by directly binding to components of mRNAs and/or miRs. Other lncRNAs function as scaffolds for chromatin-modifying factors and thus regulate epigenetics [101]. Increasing evidence suggests that lncRNA plays important roles in cardiovascular diseases, e.g., lncRNA has been shown to be downregulated after acute myocardial infarction and upregulated during the later stages of HF. This indicates that lncRNAs are associated with post-infarction cardiac remodeling and chronic HF [102]. In a study, Greco et al. found that 13 lncRNAs were significantly modulated, 10 up- and 3 down-regulated, in HF patients compared to the control group. Greco et al. also found that the lncRNAs; Cyclin Dependent Kinase Inhibitor 2B antisense RNA 1 (CDKN2B-AS1), eosinophil granule ontogeny transcript (EGOT), H19 Imprinted Maternally Expressed Transcript (H19), HOX Transcript Antisense Intergenic RNA (HOTAIR), Limbic System Associated Membrane Protein antisense RNA 3 (LOC285194), RNA Component Of Mitochondrial RNA Processing Endoribonuclease (RMRP), Ro60-Associated Y5 (RNY5), SRY-Box Transcription Factor 2 overlapping Transcript (SOX2-OT), and Steroid Receptor RNA Activator 1 (SRA1) were significantly modulated in both end- and non-end-stage HF patients [103]. Furthermore, the concentration of Zinc Finger NFX1-Type Containing 1 Antisense RNA 1 (ZFAS1), which is known as a heart-specific lncRNA, has been found to be significantly reduced within patients with AMI compared with healthy volunteers and non-AMI patients [104]. ZFAS1 expression has also been found to be increased in the myocardium of AMI patients and in cultured neonatal mouse cardiomyocytes after exposure to hypoxia for 12 h. In addition, ZFAS1 has been shown to induce intracellular Ca^2+^ overload via alteration of Ca^2+^ transit in cardiomyocytes. It has also been shown that ZFAS1 has a strong affinity for sarcoplasmic reticulum Ca2+-ATPase 2a (SERCA2a), which is a key protein involved in the maintenance of normal intracellular Ca^2+^ [105]. Furthermore, HOTAIR, which is a modulator of *HOX* gene expression, has been shown by Goa et al. to be significantly decreased in the early phase of AMI compared with control groups. Similarly, the expression of HOTAIR in cardiomyocytes exposed to hypoxia for 1, 6, and 24 h was shown to be down-regulated [106]. Studies focused on lncRNA fibroblast growth factor 9-associated factor (FAF) show that FAF exerted significantly protective effects on cardiomyocytes exposed to hypoxia. In addition, FAF has been found to regulate the expression of Fibroblast Growth Factor 9 (FGF9), which is a known protective factor in post-MI. Thus, FAF can regulate apoptosis through positively controlling FGF9 by influencing the PI3K–AKT signaling pathway [105,107]. An overview of lncRNA in ischemic heart disease is presented in Table 2.

circRNA, a type of lncRNA, consists of stable closed-ringed non-coding RNA molecules that are rich in miR-binding sites. As a result of this, circRNA can counteract the inhibitory effects on miRs on their target genes, thereby increasing the target gene expression levels. This is a mechanism known as the competitive endogenous RNA (ceRNA) mechanism. Previous findings reveal that some circRNAs are downregulated during myocardial infarction induced HF in mice, indicating a possible link between HF and circRNA [108,109]. A study focusing on the circRNA-expression profiles in peripheral blood samples from HF patients identified 56 differentially expressed circRNAs. Among the identified circRNA, hsa_circ_0097435 was found to be significantly more abundant in HF patients than in normal volunteers. Furthermore, hsa_circ_0097435 was shown to be encapsulated in exosomes. In the same study, it was demonstrated that hsa_circ_0097435 could promote apoptosis and associate with multiple miRs [110]. All these findings indicate that exosomes might have potential as biomarkers for cardiovascular disease.

## 6. Exosomes as Biomarkers

In acute pathologies, e.g., AMI, it is vital to rapidly identify and individualize the cardiac injury to optimize treatment strategies. Biomarkers are defined as measurable and quantifiable biological parameters that serve as indicators used for health and physiology assessments, such as disease risk and diagnosis [111]. A biomarker is considered good if it is easily measured and can be used as a surrogate marker for disease and its severity [112]. Ischemic related incidences are today commonly diagnosed using the cardiac biomarkers Cardiac Troponin T and I as well as Creatine-Kinase-MB [113]. Levels of Troponin I in blood have a peak 12 h after the ischemic damage to the heart and is proportional to the development of infarct size [111,114].

Shortly after the onset of injury, the heart can release characteristic exosomes of which their contents might be utilized for early diagnosis of cardiovascular diseases. Some have the capacity to replace already existing protocols, and others can be used in collaboration with classical analysis to help produce more accurate diagnoses. The discovery of exosomal miR has given rise to new possible biomarkers. An example is miR-208a, which, in a group of 66 patients (33 AMI patients and 33 non-AMI patients), was detectable in 0% of the healthy patients but detectable in 90.9% of all patients affected by AMI. The ROC curves of miR-208a between the AMI and non-AMI groups had an area under the curve (AUC) of 0.965. Furthermore, miR-208a was detected after 4 h from the onset of chest pain, which is very early compared to the appearance of detectable troponin [115]. Several other miRs have been shown to be upregulated in AMI patients, i.e., miR-208b, miR-1, miR-133a, and miR-499, but none were superior to the already existing troponin assays [116]. Moreover, in a study by Gidlöf et al., the circulating levels of miR-208b and miR-499-5p were assessed in non-MI (*n* = 88) and MI patients (*n* = 319), with an AUC of 0.82 and 0.79, respectively. In addition, the results showed that the plasma levels of miR-208b and miR-499-5p were upregulated in correspondence to the increase in the risk of death of HF, giving an indication of the prognosis, thus further solidifying miRs as possible biomarkers [117]. A study published by Matsumoto S et al. studying the expression of different miRs post-onset of AMI s (HF group, *n* = 21; control group, *n* = 65) suggests that exosome bound miRs can be used as predictive indicators of ischemic HF following AMI. Results showed that levels of p53-responsive miR-192, miR-194, and miR-34a were highly enriched in the exosome fraction in patients that developed HF post-AMI [93]. This is interesting as elevated p53 levels correlate with cardiomyocyte apoptosis and hypertrophy in end-stage human HF [118]. In addition to miRs, circRNAs have been shown to be regulated during cardiac development and failure, and may thus be suitable as potential biomarkers for HF. Moreover, the closed ring structure of circRNAs makes them more stable than linear RNA, further indicating their possibilities as useful biomarkers [119].

**Figure 4:**
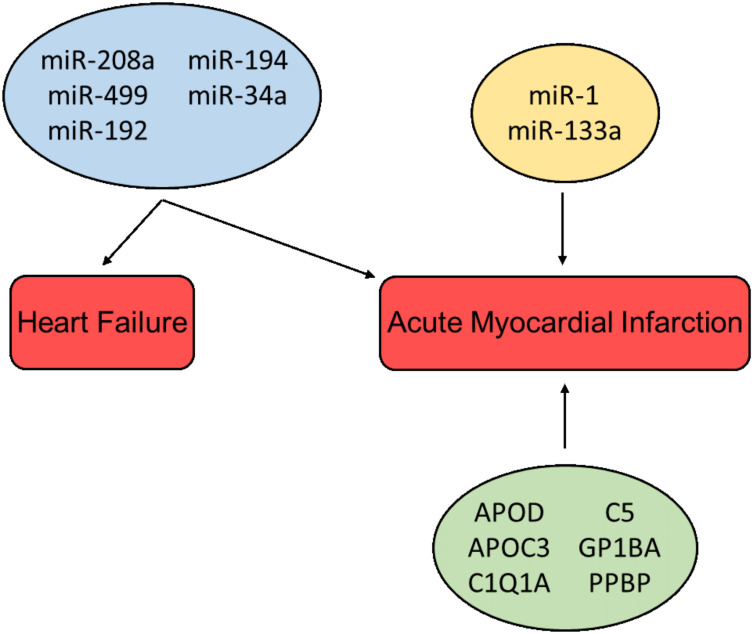
Cardioprotective factors associated with the diagnosis and prognosis of heart failure and acute myocardial infarction. miRs in the blue box are associated with multiple pathologies; miRs in the yellow box and proteins in the green box are associated with a single pathology.

In addition to non-coding RNAs, several bioactive proteins such as Apolipoprotein D (APOD) and Apolipoprotein C3 (APOC3) (lipid metabolism), C1Q1A and C5 (complement activation), Glycoprotein Ib Platelet Subunit Alpha (GP1BA), and Pro-Platelet Basic Protein (PPBP) (platelet activation pathways) have been identified in exosomes from patients suffering from MI, and thus may be used as biomarkers for myocardial injury [120]. It was shown in a study by Yu X et al. that exosomes derived from cardiomyocytes situated to hypoxic conditions mediate TNF-α production [72]. Likewise, DeJong et al. reported that in vitro hypoxia and endothelial activation stimulate upregulation of certain proteins in exosomes, e.g., fibronectin, collagen, and lysyl-oxidase-like 2 (LOXL2) [121]. Other studies have demonstrated that exosomes derived from cardiomyocytes can contain increased levels of angiotensin II type 1 receptor (AT1R), which has been shown to play an important role in maintaining blood pressure and heart function [122]. Overview of cardioprotective factors presented in Figure 4.

The composition and biological contents of exosomes vary depending on the status of the mother cells at the time of exosome biogenesis. Thus, exosomes reflect the physiological status of their parent cells. Additionally, proteins and miRs carried by exosomes are more protected from degradation and the external environment than free-floating molecules in the blood. Therefore, exosomes may function as a more reliable source of biomolecules, preventing degradation and unstable detection results. Furthermore, by assessing the different expressions of surface proteins combined with the internalized cargo of exosomes, it is possible to better identify where the signals are coming from (Organ or cell specificity), resulting in a more specific diagnosis.

## 7. Exosomes as Therapeutic Agents in Cardiovascular Disease

### 7.1. Manipulation of the Native Functions of Exosomes for Therapeutic Approaches

Currently, the most widely used methods of cardiovascular therapy are the use of liposomes and polymeric nanoparticles for drug delivery or whole-cell therapy [123]. However, the range of cardiovascular processes where exosomes have been displayed to be involved shows great potential for utilization within diagnostic as well as for discovery and applications for novel therapeutic strategies. Exosomes possess several advantages compared to existing methods: they are more stable than cells, are biocompatible, can circulate all through the body and cross the blood–brain barrier, are non-immunogenic and non-tumorigenic, and are resistant to freezing and thawing [123,124]. As earlier described, research indicates that the composition and cargo of exosomes change depending on the cell type of origin and different cellular conditions, which results in either positive or negative effects in the target cell. Therefore, as therapeutic approaches, one can either try to limit and counteract the harmful effects of some exosomes or take advantage of the beneficial effects of other exosomes.

Strategies on how to counteract the adverse effects of exosomes are based on exosome biogenesis and uptake from target cells. Trajkovic et al. found that exosome formation can efficiently be blocked by inhibiting ceramide formation using inhibitors of neutral sphingomyelinases, e.g., GW4869 [125]. Furthermore, exosome biogenesis can also be counteracted by inhibiting the interaction between syndecan proteoglycans and syntetin, which downstream interacts with programmed cell death-6-interacting protein (PDCD6IP) [126]. Additionally, MVB formation can be inhibited by using the anti-hypertensive drug named amiloride [127]. When studying the cardio-beneficial effects of exosomes, they are often isolated from CPCs, mesenchymal stem cells (MSCs), or human induced pluripotent stem cells (hiPSCs). Earlier results revealed that exosomes derived from GATA-4 overexpressing MSCs are enriched with anti-apoptotic miRs, which may promote cardiomyocyte survival during hypoxia [128]. MSCs subjected to ischemic preconditioning has been shown to produce exosomes able to reduce infarct size and fibrosis following ischemia [129]. Arslan et al. reported that exosomes isolated from MSCs could activate pro-survival signaling by increasing NADH and ATP levels following IRI [130]. Furthermore, during cardiac injury, exosomes enriched with miR-155 are secreted from activated macrophages, which results in fibroblast differentiation to myofibroblasts and increased fibrosis. Therefore, direct inhibition of miR-155 or inhibition of macrophage-specific miR-155 could be two potential therapeutic approaches for the regulation of cardiac injury [77].

Recently, Venkat P. et al. [131] published a study where they investigated if exosomes derived from human umbilical cord blood derived CD133+ cells (CD133+Exo) could improve cardiac function in type 2 diabetes mellitus (T2DM) stroke mice. Results show that CD133+Exo treatment significantly decreases cardiac tissue NOX2 and 4-HNE expression compared to T2DM-stroke control mice, resulting in decreased oxidative stress in the heart tissue. Additionally, measurements showed that expression of TGF-β, which contributes to cardiac fibrosis, was significantly lowered in the T2DM-stroke mice. Furthermore, Venkat P. et al. [131] proved that CD133+Exo treatment significantly increases the expression of miR-126 that resulted in a reduced expression of its target genes, e.g., Sprouty-related, vascular cell adhesion protein (VCAM), EVH1 domain-containing protein 1 (Spred-1), and monocyte chemoattractant protein 1 (MCP1). Furthermore, this study demonstrated that CD133+Exo treatment decreases oxidative stress, cardiomyocyte hypertrophy, and interstitial fibrosis and increases myocardial capillary density in the heart of T2DM-stroke mice [131].

To reverse post myocardial infarction injury, Gallet R. et al. sought to assess the efficacy of human CDC-derived exosome treatment using two different porcine models. Exosomes were isolated from CDC cells grown in serum-free media for 15 days and then administered either intracoronary or open-chest intramyocardially. After treatment, both scar size and scar mass decreased significantly in the exosome group compared to the control group. Furthermore, by the use of picrosirius red staining, Gallet R. et al. investigated exosome-related changes in remote fibrosis and global remodeling. Results revealed a decreased collagen content in the infarct zone as well as in more remote zones, suggesting that exosome treatment of porcine not only decreased fibrosis at the site of injection but also possess more global anti-fibrotic effects. In addition, this study showed that CDC derived exosomes was able to prevent cardiomyocyte hypertrophy associated with adverse remodeling [132].

Aminzadeh M. et al. performed a study investigating the effects of intramyocardial injection of CDC-derived exosomes into the heart of Duchenne muscular dystrophy mice (*mdx* mice). Exosomes were isolated from human CDCs conditioned in serum-free media and subjected to hypoxia (2% O_2_; default condition) overnight. The results revealed that intramyocardial injection of CDC derived exosomes could improve left ventricular function and volume. Moreover, the addition of CDC exosomes causes major changes in the gene expression of proteins related to inflammation, oxidative stress, and mitochondrial integrity. CDC exosomes activate the Nuclear Factor Erythroid 2 Like 2 (NRF2) antioxidant pathway, which increases the protein levels of phosphorylated AKT, and NRF2, as well as downstream gene products (heme oxygenase 1 (HO-1), Superoxide dismutase 2 (SOD-2), and Glutamate-Cysteine Ligase Catalytic Subunit (GCLC)), ultimately resulting in attenuated oxidative stress. Furthermore, histologic analyses revealed that CDC treated *mdx* hearts possessed much less fibrosis. Likewise, CDC exosome treatment reversed activation of Nuclear factor-κB (NF-kB) and decreased the number of inflammatory cells in *mdx* hearts [133].

A recent study published by Dougherty et al. indicates that the therapeutic potential of human CPC-derived exosomes can be improved by culturing the human CPCs (hCPCs) in a low-oxygen (i.e., physoxic 5% O_2_) microenvironment. More specifically, the study showed that the exosomal cargos released during physoxic conditions are very potent for promoting angiogenesis. Furthermore, hCPCs cultured under physoxic conditions were able to maintain normal cell morphology and cardiac marker expression. In addition, hCPCs cultured at 5% O_2_ showed an increased exosome secretion compared to those cultured under normal and hypoxic conditions. With this study, Dougherty et al. elucidated the potential of physoxic culturing of hCPCs, as well as how it may play a crucial role in myocardial repair applications. Further studies on physoxic culturing and the functional outcome of hCPC derived exosomes seem to be a promising direction towards improving cardiac repair [134,135].

### 7.2. Therapeutic Potential of Non-Native Exosomal Cargo

In addition to the natural cardioprotective effects of exosomes, several research groups have tested the therapeutic potential for packing exosomes with non-native [136]. For the purpose of crossing the phospholipid membrane of exosomes, multiple viable loading techniques exist. Exosomal membrane permeability can be altered by external electric field application resulting in hydrophilic pore formation and increased permeability, thereby allowing for the passage of DNA, RNA, and chemicals into the exosome. This process is called electroporation/electropermeabilization [137]. Multiple studies have shown successful loading of RNA into exosomes via electroporation [138,139]. Alvarez-Erviti et al. managed to load dendritic cell derived exosomes with siRNA via electroporation and demonstrated efficient delivery of siRNA to target neural cells in vitro as well as in vivo [140]. Despite several demonstrations of successful exosome loading via electroporation, it is described that the technique favors extensive siRNA aggregation, which overestimates the amount of loaded siRNA into extracellular vesicles [141]. Furthermore, exosomes can also be loaded with therapeutic cargo by overexpressing a certain gene in exosome-donor cells or treat cell lines with a drug of interest that will later be gently enveloped into vesicles based on the process of exosomal biogenesis [136]. Protein, mRNA, miR, and siRNA have been successfully endogenously inserted via prior vesicle-secreting cell transfection. In a study by Kanada et al., HEK293FT donor-cell were transfected with plasmid mRNA. The study confirmed that mRNA could successfully be loaded and transferred by exosomes [142]. Furthermore, recent reports [143,144] have demonstrated that incubation or treatment of exosome parental cells with cargo or drug of interest can result in substance incorporation into a vesicle during extracellular vesicle biogenesis. An alternative utilized exosome loading technique is called sonication, which is based on the sonoporation phenomenon where exosomes are situated to low-frequency ultrasound, which induces cavitation bubble formation. The bursting of microbubbles produces exosome membrane pores, allowing for the crossing of genetic material into the exosomes [136,145]. Lamichhane et al. managed to successfully apply sonication for siRNA loading into extracellular vesicles and showed successful delivery of siRNA to recipient cells for Human epidermal growth factor receptor 2 (HER2) gene silencing in breast cancer [146].

Despite the documented potential of exosomes in protecting the heart and other organs, no optimal way of exosome isolation and purification for characterization exists. This is crucial before exosomes can be delivered to ischemic areas in clinical application.

## 8. Exosome Isolation and Characterization

The major obstacle in the clinical utilization of exosomes is the lack of consistent and reliable methods to isolate a pure exosome population without damaging their integrity. Techniques used for exosome isolation must be highly efficient and possess the capability to isolate exosomes from various tissue samples. Exploiting the physiological- and biochemical properties of exosomes, e.g., shape, density, size, and surface proteins, has given rise to several isolation techniques [147,148]. The most often utilized method of exosome isolation is ultracentrifugation, which consists of a series of centrifugation cycles of different centrifugal force and duration. Ultracentrifugation isolate exosomes based on density and size differences compared to other components in a sample [149]. A more efficient method than ultracentrifugation is ultrafiltration. Ultrafiltration isolate exosomes using membrane filters with defined molecular weight or size exclusion limits [150]. Moreover, several isolation techniques, e.g., microplate-based enzyme-linked immunosorbent assay (ELISA), utilize the immunoaffinitive interactions between exosomal membrane-bound proteins and their antigen, or specific interactions between exosomal receptors and ligands. In addition, several commercial isolation kits, e.g., ExoQuick™ and Total Exosome Isolation™ (TEI), are now available [151]. Over the past decade, impressive progress has been made in the development of exosome isolation techniques, which has contributed to decoding the mystery of exosomes [152]. However, the aforementioned methods have major drawbacks such as aggregation, impurities, disruption of biological integrity, and low recovery yields. Moreover, they are labor-intensive and time-consuming. It is worth noting that efforts to standardize the method of collection, centrifugation, and transport are being made [148]. Moreover, each method for exosome isolation and purification has its pros and cons, highlighting the need for careful consideration in regard to the research purpose. An overview of isolation methods that can be found is presented in Table 3.

Furthermore, when isolating a pure exosomal population, one must be able to identify the exosomes from the other extracellular vesicles in a sample. Generally, what separates exosomes from other EVs like microvesicles and apoptotic bodies are their size, biogenesis pathway, and content [153]. Microvesicles are larger in size than exosomes, ranging from 200 nm to 1 µM. Microvesicles are formed by the outward budding of the plasma membrane; thus, they are shed directly from the plasma membrane [154]. Proteomic analysis of exosomes has identified several protein markers, including heat shock proteins (HSP70 and HSP90), tetraspanins (CD9, CD63, and CD81), actin, annexins, glyceraldehyde-3-phosphate dehydrogenase, and enolase. Exosomes also contain molecules involved in MVB biogenesis, e.g., Alix, TSG101, and Rab-proteins [155]. There are several characterization and validation methods that have been developed to analyze exosome purity and to quantify exosomal cargos. Some of these methods are scanning electron microscopy (SEM), transmission electron microscopy (TEM), nanoparticle tracking analysis (NTA), atomic force microscopy (AFM), dynamic light scattering (DLS), and fluorescence-activated cell sorting (FACS) [156]. An overview of characterization methods is presented in Table 4.

For exosome study of the ischemic heart, a measurement can be performed from blood, pericardial fluid, or lymphatic samples in an in vivo setting and from perfusate samples collected from the heart during Langendorff perfusion and from media of ischemic cardiomyocyte models in culture. When isolating exosomes from the blood, it is important to know that pre-analytical procedures can have a large impact on the measurements, e.g., clotting of the blood can increase the number of extracellular vesicles in blood 10-fold. Furthermore, depending on what is most important for the study, exosome purity or overall yield, exosomes should be isolated from plasma or serum, respectively. In order to reduce sample viscosity, it is recommended to dilute blood plasma or serum at least 2× in Ca^2+^-free PBS prior to centrifugation. Shortly after centrifugation, the desired sample can be carefully collected and stored at −80 °C prior to characterization [163]. Additionally, exosomes isolated from the pericardial fluid may provide useful information about cardiac health [164]. Unfortunately, there is as of yet no consensus on what the best method for isolation of exosomes from the pericardial fluid is [163]. For the isolation of exosomes from tissue cultured in media, the typical source of contamination comes from the use of fetal bovine serum (FBS). This is because FBS contains a large number of lipoproteins as well as exosomes and other vesicles [165]. Therefore, serum-free conditions can be used. High-Performance Liquid Chromatography (HPLC) has been proven to be quite successful for the purification of exosomes from cultured tissue. For the isolation, any of the aforementioned techniques can be used [163]. As mentioned earlier, exosomes can be characterized by specific markers, such as tetraspanin proteins (e.g., CD9 and CD81), HSP70, and flotillin-1. Moreover, exosomal surface marker expression and internalized cargo can be used to identify its cell-type of origin, including cardiac cells, as discussed above, and also reviewed elsewhere [166,167].

Finally, to assess the functional interaction or uptake of exosomes to recipient cells, which is essential for direct verification of exosomes as the carrier of therapeutics, the exosomes can be labeled with lipophilic fluorochromes, or paired with fluorescence protein tags, e.g., green fluorescence protein [163].

## 9. The Future of Exosomal Cardiovascular Treatment

As the interest in exosomes is increasing, more and more of their useful applications are elucidated. Understanding the exact role of exosomes and especially their cargo is vital for exploring how cells are able to communicate adaptive processes to other cells and organs, such as initiating cardioprotective effects as well as repairs and adaptive regeneration during and following ischemic damage. Furthermore, the study of exosomes from the ischemic heart may reveal important communication and signaling mechanisms for local release in the heart as well as distant tissue such as skeletal muscle and bone marrow.

There are several potential opportunities for future applications of the use of exosomes within the cardiovascular field: either as biomarkers for diagnostics purposes or for estimating the prognosis pathological cardiac development, as therapeutic carriers of biomolecules, or for the discovery of novel targets of treatment guided by the exosomal content. For instance, several studies have proven that levels of the specific exosomal miR rise in patients subjected to cardiac damage and that some miRs are upregulated in correspondence to the increase in the risk of death, indicating that exosomal miRs has the possibility to both indicate cardiac damage and severity. Although the results obtained are promising, the underlying mechanism of secretion and sorting of exosomal miR from cardiac cells are yet to be well characterized. Especially, the relevance of extracellular miRs and their potential off-site target effects in different pathophysiological states needs extensive investigation before potentially considering the transition to clinical applications. Furthermore, despite the promising results assessing exosomes as possible biomarkers for IHD, their accuracy and efficacy are, however, still at a premature stage to replace traditional biomarkers, e.g., Troponin T and I. As of now, exosomes as biomarkers are best suited as a supporting factor in IHD detection and prognosis. However, in recent years, advancements in the characterization of exosomes hold a promise for more efficient and technically improved isolation and characterization, exosomes will provide the opportunity for quicker and more specific diagnostics in the future.

It is important to note that exosomes have been shown to induce both negative or positive cardiovascular effects, depending on the situation and their source of origin. Especially, considering that exosomes can be released by a range of different cells, including, e.g., tumor cell, immune cells, cardiac cells, and bodily fluids, it will be important for future research to enable technological platforms that can trace back the exosomes to the cell types where they were released. The adverse effects of exosomes can be counteracted by blocking exosome formation and secretion or uptake in target cells. By isolating exosomes from stem cells of different origins, it has been demonstrated that exosomes can promote cardiomyocyte survival and reduce infarct size and fibrosis following ischemic incidences. Furthermore, in addition to being natural carriers of cardioprotective biomolecules, exosomes seem to hold great potential as vehicles for targeted drug delivery and therapy. However, despite the promising potential for the use of exosomes, rapid and efficient isolation of exosomes has been proven difficult due to the complexity of biological samples, considerable overlap between other extracellular vesicles, heterogeneity of exosomal surface and content, as well as the exosome kinetics. Furthermore, since exosomes are susceptible to change, setting up scalable and reproducible isolation processes can be difficult. It is therefore important to carefully define the different populations of exosomes with highly efficient exosome isolation and characterization techniques before utilizing them in any field. Unfortunately, standardized, cost-efficient methods that produce a high yield of exosomes with intact biological integrity and function remains insufficient. The recent introduction of standing surface acoustic waves (SSAW) presents a possibility for label-free separation of particles in a range of sizes with external control, thus potentially enabling continuous isolation of exosomes without fixation or induced exosomal damage. Developments in this field could therefore represent a major step forward in the clinical utilization of exosomes [168].

## 10. Conclusions

This review discusses the role of exosomes in cardiovascular disease, and especially in the context of ischemic heart disease, ischemia reperfusion injury, and ischemic cardioprotection. Exosomes with origin from cardiac cells hold unprecedented opportunities for future applications either as biomarkers for diagnostics purposes, for estimating the prognosis of pathological cardiac development, as therapeutic carriers of biomolecules, or for the discovery of novel targets of treatment guided by the exosomal content. Furthermore, we highlight the limitations of current exosome isolation and characterization techniques, which remains at suboptimal levels before considering a transition to clinical application for treatments of cardiovascular diseases. For future research, a combination of nanotechnology, fluid mechanics, biotechnology, and medicine to develop better separation, purification, characterization, and treatment techniques hold potential for further advancements of exosome-based treatment against cardiac ischemia.

## Figures and Tables

**Table 1:** MicroRNA (miR) in ischemic heart disease.

miR ID	Change in Expression	Pathology	Reference
miR-18a	↓	HF	[89]
miR-27a	↓	HF	[89]
miR-30e	↓	HF	[89]
miR-26b	↓	HF	[89]
miR-199a	↓	HF	[89]
miR-106a	↓	HF	[89]
miR-652	↓	HF	[89]
let-7i	↓	AHF	[99]
miR-18a	↓	AHF	[99]
miR-18b	↓	AHF	[99]
miR-223	↓	AHF	[99]
	↓	AMI	[89]
miR-301a	↓	AHF	[99]
miR-652	↓	AHF	[99]
miR-423	↓	AHF	[99]
miR-21	↑	SHF	[91]
	↑	AMI	[89]
miR-1	↓	SHF	[91]
	↑	AMI	[89]
miR-1254	↑	CHF	[91]
miR-1306	↑	CHF	[91]
miR-133	↑	AMI	[89]
miR-29b	↑	AMI	[89]
miR-192	↑	AMI	[89]
miR-194	↑	AMI	[89]
miR-34a	↑	AMI	[89]
miR-208	↑	AMI	[89]
miR-499	↑	AMI	[89]
miR-423	↑	AMI	[89]
miR-126	↑	AMI	[89]
miR-134	↑	AMI	[89]
miR-328	↑	AMI	[89]
miR-486	↑	AMI	[89]
miR-106	↓	AMI	[89]
miR-197	↓	AMI	[89]

HF, heart failure; AHF, acute heart failure; AMI, acute myocardial infarction; SHF, systemic heart failure; CHF, chronic heart failure.

**Table 2:** Long non-coding RNA (lncRNA) in ischemic heart disease.

lncRNA	Pathology	Reference
CDKN2B-AS1	HF	[103]
EGOT	HF	[103]
H19	HF	[103]
HOTAIR	HF	[103]
LOC285194	HF	[103]
RMRP	HF	[103]
RNY5	HF	[103]
SOX2-OT	HF	[103]
SRA1	HF	[103]
ZFAS1	AMI	[104]
HOTAIR	AMI	[106]

HF, heart failure; AMI, acute myocardial infarction.

**Table 3:** Available exosome isolation methods.

Isolation Method	Potential Advantages	Potential Disadvantages	Technical Principles	References
Ultracentrifugation	Produces highly enriched EV fractions. Large Sample capacity Reduced cost.	Isolation of impurities Low reproducibility Low RNA yield Damage of exosomes Low throughput Labor-intensive Time-consuming	Separation of particles based on their density, size and shape by high-speed centrifugation.	[148,149]
Ultrafiltration	Fast. No special equipment needed. Direct RNA extraction possible.	Moderate purity exosomes Exosome shear stress Lower-exosome yield	Isolation exclusively based on the size difference between particles.	[148,150]
ELISA	Excellent for isolation of specific particles. High sample purity.	High costs Low capacity and yield	Isolation based on specific interaction between particle membrane-bound antigens immobilized antibodies.	[148]
Commercial Isolation Kits	Easy to use. Large and scalable sample capacity. No special equipment needed.	Co-precipitation of impurities Time consuming	Altering particle solubility by the use of water-excluding polymers.	[150,152,153]

**Table 4:** Methods for characterization of exosomes.

Characterization Method	Detectable Size Range	Advantages	Disadvantages	Technical Principles	References
SEM	>5 nm	High-resolution imaging.	Complex sample preparation. Requires fixation and drying of the sample.	Characterize particle topography by detecting secondary electrons with a focused electron beam.	[157]
TEM	>5 nm	High-resolution imaging.	Complex sample preparation. Requires fixation and drying of the sample.	Characterize particles. Based on the transparency of their features to an electron beam.	[157,158]
NTA	50–1000 nm	Fast assessment of size distribution and concentration of particles.	Subject to statistical uncertainties.	Determine the size and density of particles in suspension based on their Brownian motion in a static solution.	[157,159]
AFM	>5 nm	Sample labeling not needed. Offers unique information, e.g., particle stiffness and elasticity.	Low throughput. Technically demanding.	Characterize particle topology with nanometer resolution by scanning the area with an extremely sharp tip.	[157,160,161]
DLS	5–2000 nm	Simple and quick analyses.	Limited utility in the analysis of minimally processed biofluids.	Determine the size distribution of particles by analyzing temporal intensity fluctuations of laser light.	[157,162]
FACS	>300 nm	Able to identify specific EV subpopulations.	Low detection sensitivity for EV.	Characterize size and particle subpopulations by scattered and fluorescent light from hydro-dynamically focused particles.	[157]

## Data Availability

Not applicable.
